# Potential resilience treatments for orangutans (*Pongo* spp.): Lessons from a scoping review of interventions in humans and other animals

**DOI:** 10.1017/awf.2023.97

**Published:** 2023-12-04

**Authors:** Lelia Bridgeland-Stephens, Susannah KS Thorpe, Jackie Chappell

**Affiliations:** School of Biosciences, University of Birmingham, Birmingham, UK

**Keywords:** animal welfare, captivity, great ape, orangutan, resilience treatment, stress inoculation

## Abstract

Wild orangutans (*Pongo* spp.) rescued from human-wildlife conflict must be adequately rehabilitated before being returned to the wild. It is essential that released orangutans are able to cope with stressful challenges such as food scarcity, navigating unfamiliar environments, and regaining independence from human support. Although practical skills are taught to orangutans in rehabilitation centres, post-release survival rates are low. Psychological resilience, or the ability to ‘bounce back’ from stress, may be a key missing piece of the puzzle. However, there is very little knowledge about species-appropriate interventions which could help captive orangutans increase resilience to stress. This scoping review summarises and critically analyses existing human and non-human animal resilience literature and provides suggestions for the development of interventions for orangutans in rehabilitation. Three scientific databases were searched in 2021 and 2023, resulting in 63 human studies and 266 non-human animal studies. The first section brings together human resilience interventions, identifying common themes and assessing the applicability of human interventions to orangutans in rehabilitation. The second section groups animal interventions into categories of direct stress, separation stress, environmental conditions, social stress, and exercise. In each category, interventions are critically analysed to evaluate their potential for orangutans in rehabilitation. The results show that mild and manageable forms of intervention have the greatest potential benefit with the least amount of risk. The study concludes by emphasising the need for further investigation and experimentation, to develop appropriate interventions and measure their effect on the post-release survival rate of orangutans.

## Introduction

All three orangutan species (*Pongo* spp.) are critically endangered in the wild and face substantial population decline (Ancrenaz *et al.* 2018; Singleton *et al.* 2018). As the only great ape species endemic to the islands of Borneo and Sumatra, orangutans are threatened by a combination of factors, including habitat loss, degradation, and fragmentation (Wich *et al.* 2015, 2016). Many are deliberately killed as a by-product of habitat conversion, or as a result of human-wildlife conflict, which can lead to infants being captured and trafficked in the illegal wildlife trade (Ancrenaz *et al.* 2018; Singleton *et al.* 2018). Since pioneering efforts in the 1960s, a number of rehabilitation centres have been established across Borneo and Sumatra, with the aim of rescuing injured and/or trafficked orangutans and providing them with long-term rehabilitation (Russon 2008). The purpose of this is to prepare capable individuals for release back into the wild, and to provide lifelong care for those who are unable to be released, for example if they have a severe physical disability, or an inability to acquire the necessary skills for independent living. As many orangutans in rehabilitation are infants rescued from the illegal wildlife trade (Russon 2008), these individuals will spend a large proportion of their development in rehabilitation centres before they are old enough to be released back into the wild. Although other great ape species are released back into the wild, this process is more established for orangutans than the other great apes. This is partly because the Indonesian government mandates the release of all orangutans where possible (Sherman *et al.* 2020). Therefore, this review focuses on orangutan rehabilitation due to the more urgent need considering ongoing orangutan releases, and the fact that established rehabilitation and release practices for orangutans are already in place.

Wild orangutan infants stay with their mothers until they are around six to eight years old (van Noordwijk & van Schaik 2005), learning all the essential skills they need to survive as an adult. Life in the wild for the world’s largest arboreal mammal (Cant 1992) is extremely demanding. Released orangutans will need to face challenges such as the physical and cognitive requirements of travelling through the forest canopy and building appropriate nests (Thorpe *et al.* 2007; van Casteren *et al.* 2012; Halsey *et al.* 2017), coping with unpredictable food scarcity (Knott 1998), encountering unfamiliar or difficult-to-process food (Jaeggi *et al.* 2010), interacting with other orangutans, and managing potential conflict with humans and other dangerous animals (Meijaard *et al.* 2011; Chappell & Thorpe 2022). In addition, when female orangutans breed successfully after being released, they must face all these challenges while providing their infants with extensive care until weaning, without having had the opportunity to experience species-typical maternal care from their own mothers.

As many orangutans in rehabilitation centres are orphaned at a young age, they are completely dependent on the support and care of human caretakers, and being able to learn from their peers, to help them acquire these life skills and prepare for independent living in the forest. Despite the extensive current efforts of rehabilitation centres to help orangutans develop each of these skills, there are continuing issues with survival after release into the wild (Russon 2008; Utami-Atmoko *et al.* 2017; Basalamah *et al.* 2018). One survey found that the reported survival rates of released orangutans range from 20 to 80%, with an estimated average 40% survival rate (Russon 2008). Therefore, even if orangutans appear to exhibit individual life skills while in rehabilitation, this does not necessarily translate into the capacity for independent survival. This indicates that there is a missing piece of the puzzle, and suggests that there may be a broader, less tangible, factor underpinning the successful adaption of orangutans to the wild.

In a survey of Orangutan Veterinary Advisory Group (OVAG) members (n = 43), 93% of respondents (40/43) agreed that an orangutan’s ‘drive to survive’ was an important factor in deciding whether to release orangutans back into the wild (unpublished OVAG questionnaire, L Bridgeland-Stephens 2020). Although OVAG was established with the primary purpose of providing support and advice to vets working with orangutans, its members and conference attendees (who were consulted in this survey) include a wide range of professionals working in orangutan rehabilitation, including centre managers, animal caretakers, and scientists. Participants described orangutans with the ‘drive to survive’ as having the motivation and skills to meet their own well-being needs, successfully adapt to the forest environment, and show durability in overcoming challenges. Anecdotally, participants describe finding that some individuals just give up, while others remain engaged with their new environment. Each of these factors is constructive in ensuring survival after orangutans have been released into the wild. This idea of individual variation in orangutans’ ‘drive to survive’ arguably overlaps with the concept of ‘resilience’, which can be defined as a successful adaptation or competence despite stress or trauma (Egeland *et al.* 1993). In humans, a lack of resilience can be expressed in many ways, for example passive or helpless behaviour, a short attention span, or disregarding and even dismantling solutions to a problem which at first seem to be successful (Janoff-Bulman & Brickman 1982). Resilience influences the way in which events or problems are approached, and can be influenced by factors such as temperament, positive emotions, self-esteem, planning skills, and supportive environments (Fletcher & Sarkar 2013). Traits indicative of resilience include resourcefulness, flexibility, high levels of activity, optimism (see S1 Appendix in Supplementary material), and curiosity (Block & Block 1980).

There is a lack of existing knowledge regarding resilience in non-human great apes. However, it seems likely that resilience, and the ability to ‘bounce back’ from stress, would be important for orangutans in rehabilitation centres. Resilience may also underpin the ‘drive to survive’, highlighted by OVAG members as an important aspect of successful orangutan releases. All orangutans in rehabilitation will have experienced at least one major life stressor, in being captured and then travelling to the rehabilitation centre. In addition, prior to rehabilitation, many will have witnessed the death of their mother, experienced physical injuries from humans or other orangutans, poor living conditions in small cages, or emaciation from lack of food (Sherman *et al.* 2020). They are likely to continue to experience stressors during their time in rehabilitation, for example conflict with conspecifics, veterinary interventions such as surgeries or routine health checks, being near dominant males (Mitra Setia & van Schaik 2007; Great Ape Taxon Advisory Group 2018; Kunz *et al.* 2021), and moving to unfamiliar environments (e.g. pre-release islands). As well as overcoming stressors during rehabilitation, it seems likely that psychological resilience would be particularly important when orangutans are released, so that they have the capacity to adapt to challenges related to life in the wild. Low levels of resilience could explain why some orangutans appear to simply ‘give up’, despite their skillset, because persistence is an important aspect of resilience (Grotberg 1995; Brown 2015). In this context, ‘give up’ means not striving to meet one’s own well-being needs, thereby increasing the likelihood of serious harm or death. Behaviours that indicate a striving to meet well-being needs include actively seeking food and avoiding predators, building appropriate nests, establishing a range, and travelling through the canopy rather than across the ground. Resilience may be an important missing piece of the puzzle, potentially underpinning the chances of a successful release by providing orangutans with a more flexible and generalised ability to ‘bounce back’ from previously unencountered challenges, rather than having to rely upon specialised, situation-specific skills.

In humans, resilience is considered to be a malleable, rather than fixed, characteristic (Fletcher & Sarkar 2013). Similarly, although little is known about orangutan resilience, 70% of OVAG respondents (n = 30/43) felt that an orangutan’s ‘drive to survive’ could be influenced through the rehabilitation process (unpublished OVAG questionnaire, L Bridgeland-Stephens 2020). However, despite the potential importance of psychological resilience on the success of rehabilitation and release in orangutans, this factor has not yet been addressed scientifically. In order to begin filling this gap in scientific knowledge, this review will draw together human and animal research on resilience interventions, to establish an understanding of the foundations of resilience, and to identify possible resilience-promoting treatments for orangutans. ‘Resilience treatments’ are defined here as interventions designed to influence one or more attributes of resilience in an individual. In reviewing the existing literature, the word ‘treatment’ is taken to mean an experimental condition or intervention, including treatments expected to have a negative impact. It is important to understand the potential negative effects of specific interventions in order to avoid replicating them and thereby causing further harm. It should be noted that resilience is a latent variable and can only be measured or influenced indirectly. Therefore, interventions designed to promote resilience are likely to overlap and inter-relate with tools to measure resilience, as both are proxies of ‘true’ resilience.

The purpose of this review is to outline key recommendations for practical interventions that can be used to promote resilience in orangutans in rehabilitation centres. Although the findings of this review are more immediately useful for orangutans in rehabilitation centres, they also have relevance for orangutans in zoos and sanctuaries, as well as for other species of captive great apes. Due to the lack of research into great ape resilience interventions, this review relies upon existing research on humans and other species, largely rodent studies. Due to the close phylogenetic relationship and cognitive overlap among great ape species, and the resulting ‘psycho-socio-biological continuity’ (Brüne *et al.* 2006), it is not unlikely that many of the main factors underpinning resilience in humans are shared by other great apes. There is a longstanding tradition of comparative research on aspects of psychology, for example theory of mind (Premack & Woodruff 1978), attachment theory (Harlow 1958), learned helplessness (Overmier & Seligman 1967), and psychopathology (e.g. Fabrega 2002). In addition, although rodents are not closely related to great apes, rodent studies have long been used as a starting point for pre-clinical medical research due to similarities in anatomy, physiology, and genetics (Bryda 2013) (though for limitations of animal studies in human psychology, see Shapiro 1998). In conjunction, research on human and non-human animals will help to build a picture of factors underpinning resilience and possible treatments. However, the findings of this scoping review should only be interpreted as starting points for cautious future research into great ape resilience treatments. Section one will address human resilience treatments. Although most of these methods are not applicable to great apes, it is important to understand the essential elements of resilience and identify common themes that underpin successful resilience treatments. Therefore, this section will draw out key concepts that have the potential to be translated into interventions for orangutans. Section two will summarise the most common resilience treatments in studies of non-human animals, hereafter referred to as animals. The animal resilience treatments described in section two all overlap with at least one theme described in the human resilience section. Each treatment type will be critically analysed to evaluate its relevance for orangutan rehabilitation centres, and practical suggestions will be made for integrating similar techniques into appropriate resilience treatments for orangutans.

## Materials and methods

An electronic search was conducted on 7^th^ February 2021 using three search engines: ProQuest Biological Science Collection, Scopus, and Web of Knowledge. Boolean operators were used for the following search terms: “psychological resilience” OR “resilience interventions” OR “resilience program” OR “stress inoculation” OR “stress immunity” OR “foster resilience”; and “psychological resilience” AND “animal”. The language filter was set to English, and the sources included were books, conference papers and proceedings, dissertations and theses, government & official publications, reports, scholarly journals, and working papers. There was no exclusion based on publication date.

The initial search returned 1,680 results, reduced to 1,241 articles of potential relevance after removing duplicates. The reference lists of the animal articles were also searched for relevant articles, as a greater diversity of treatment type was identified in the animal literature compared to the human literature. Only resources which had institutional access were used, excluding 20 articles from the final list. Irrelevant articles, and those which related to a very specific aspect of resilience, for example in a particular activity or relating to a particular illness or pain threshold, were removed in favour of articles with more general relevance. Studies on prenatal stress, and studies proposing medicinal aids to resilience, were excluded. Finally, only interventions which were targeted at building individual resilience (rather than family resilience, or group resilience) were included, even if the resilience interventions were provided in a group setting. The search and filtering process was carried out by the lead author and resulted in 63 human papers and 240 animal papers. An additional search of animal articles was carried out on 11^th^ May 2023, using the same search terms and filtering criteria as before, to ensure that the literature was up-to-date. This returned 202 results, and, after removing duplicates (n = 1) and articles that did not meet the criteria (n = 175), resulted in a further 26 relevant animal studies, bringing the total number of animal studies to 266. Due to the breadth of fields drawn together in this review, a glossary for lesser-known terms is provided in S1 Appendix in the Supplementary material.

## Section one: Human resilience treatments

The human resilience studies encompassed a broad range of age groups and spanned five continents (Asia, Australia, Europe, and North and South America). An overwhelming majority of the treatments used talking therapies, in a group or individual setting. The in-text descriptions of the content of each resilience programme were coded into different categories and then collated into broader recurring themes, listed in Table 1. Many of these themes match those described by OVAG members as important factors in an orangutan’s ‘drive to survive’; for example, flexibility, hardiness, self-efficacy, independence, social skills and interest in conspecifics, physical activity, problem-solving, and the ability to overcome stress (unpublished OVAG questionnaire, L Bridgeland-Stephens 2020).

**Table 1.** Common themes in human resilience interventions

Type of treatment	Number of studies
Stress inoculation	31
Emotional intelligence	25
Social skills, peer support	23
Self-awareness, self-reflection	22
Optimism, positive affect, positive thinking	21
Cognitive skills	19
Self-confidence, self-esteem	19
Relaxation, meditation, mindfulness	18
Self-control, behavioural inhibition	17
Agency, self-efficacy, independence	15
Problem-solving	14
Planning	11
Exercise, physical activity, healthy lifestyle	9
Persistence, hardiness	9
Goal orientation	7
Flexibility	7
Other	5

‘Number of studies’ includes all studies which mention each theme; some studies include multiple themes (see S1 Appendix in Supplementary material for definitions of terms).

Interventions included training in one or more of these themes, with a mean of 6.5 different themes per treatment and a range of 1–15. Studies can be broadly divided into those based on a type of cognitive behaviour therapy called Stress Inoculation Therapy (see S1 Appendix; Supplementary material) (Meichenbaum 1985), further detailed in *Stress inoculation* below, and those using alternative methods. More than 80 different types of questionnaire were used to measure aspects of resilience, shown in S2 Appendix (Supplementary material). These were either carried out by an experimenter/professional or self-administered.

### Resilience interventions

Several interventions were found to significantly increase resilience (Steinhardt & Doblier 2010; Peng *et al.* 2014; Rogerson *et al.* 2016; Pluess *et al.* 2017; van Agteren *et al.* 2018; Joyce *et al.* 2018; Henshall *et al.* 2020), particularly in individuals who initially had low levels of resilience (Peng *et al.* 2014; van Agteren *et al.* 2018). In applying a resilience training programme to female prisoners, which focused on mindfulness techniques, ‘positive psychology’ (see S1 Appendix; Supplementary material), and cognitive behavioural therapy, Lo *et al.* (2020) identified a greater beneficial effect of the programme for long-term prisoners than prisoners awaiting release. This is encouraging for orangutans unable to be released into the wild who need to cope with pressures connected with long-term life in captivity, for example relative space limitations and lack of environmental complexity. Although there are clear differences between human prisoners and animals in long-term captivity, particularly regarding the intentions and causes underlying each situation, there are arguably similarities in the physical limitations and psychological stress (Morgan & Tromborg 2007) resulting from long-term life in captivity. In a resilience training programme with similar methods to Lo *et al.* (2020), Smith *et al.* (2018) found that the longer participants engaged with resilience training, the greater the benefit they experienced. This indicates that it is beneficial to integrate resilience-building interventions throughout the whole rehabilitation period, to maximise the potential benefits. Although mindfulness and positive psychology themselves are not applicable to orangutans (as with all forms of talking therapy), it seems likely that their effectiveness in building resilience is related to the individual mechanisms of action of these methods, e.g. increasing relaxation and optimism (Rygula *et al.* 2015; Roelofs *et al.* 2016). Therefore, their inclusion is still relevant in terms of identifying aspects of resilience treatments which can be effective.

A range of studies that focused on talking therapies found benefits in measures of specific aspects of resilience, including the ability to cope, proactivity, self-esteem, confidence, and lower levels of stress, depression, anxiety, inflexibility, and negative or suppressed emotions (Steinhardt & Doblier 2010; Peng *et al.* 2014; Gallegos-Guajardo *et al.* 2015; Pluess *et al.* 2017; van Agteren *et al.* 2018; Joyce *et al.* 2018; Foster *et al.* 2018; Henshall *et al.* 2020; Kozina 2020; Lo *et al.* 2020; Akeman *et al.* 2020). Pluess *et al.* (2017) identified long-lasting beneficial effects in schoolchildren who had participated in a resilience treatment programme, when tested six and twelve months after the treatment. Neither Chandler and Roberts (2015) nor Delaney *et al.* (2016) found a statistically significant difference between resilience treatment and control groups, although both studies had a relatively low number of participants (n = 28 and 40, respectively) and benefits were self-reported by participants in the form of written reflections or in-depth interviews. Reported benefits included building personal strengths, creating supportive connections with others (Chandler & Roberts 2015), learning stress-relieving techniques, and sharing experiences of stress with others (Delaney *et al.* 2016).

These results suggest that it is possible for humans to build skills and attributes that contribute to resilience and protect against stress. However, the treatments above are all talking therapies, and non-linguistic methods are required for orangutans. In addition, resilience therapies in humans may have an underlying benefit by providing a degree of social support, same-species contact, and the feeling of ‘being understood’, and these benefits may not translate in a straightforward way for orangutans. However, the most common themes in human resilience treatments, shown in Table 1, offer a starting point for the design of orangutan interventions which incorporate one or more of these resilience themes.

### Stress inoculation

#### Stress inoculation therapy

Stress Inoculation Therapy (SIT) (Meichenbaum 1985) involves a form of cognitive behavioural therapy, and is based on the theory that behaviour and feelings are determined by individual perceptions, and can therefore be cognitively reframed. SIT is intended to break the ‘stress cycle’ of negative reactions and ineffective responses to stressful events by teaching a range of coping skills and behaviours. The three-stage process involves patients reconceptualising their responses to specific stressors, before learning coping skills and applying these to imaginary (role-played) and real-life stressors (Meichenbaum & Cameron 1983).

SIT was used by a treatment in 27 of the papers reviewed, and several randomised controlled trials identified beneficial effects (Law *et al.* 1994; Szabo & Marian 2012; Varker & Devilly 2012; Hourani *et al.* 2016; Navaee & Kaykha 2019), with some positive effects still present two years after the intervention (Hourani *et al.* 2016). However, in the context of orangutan rehabilitation, the cognitive reframing element of SIT would be extremely difficult to achieve in a non-linguistic form and may be counterproductive in blunting responses to stressors, for example by promoting the inhibition of appropriate reactions to stressful events during the ‘skills acquisition’ phase of the treatment (Meichenbaum & Cameron 1983). It is important for caretakers to be able to identify stressors for orangutans by reading their behaviour, to avoid further harm. This is already a challenge, as non-human primates are adept at masking overt expressions of pain and illness (Pelsker & Mayer 2008). SIT has also received criticism for moving the locus of control away from the patient (towards the therapist), and for framing certain responses to stress as ‘irrational’ (Hurley *et al.* 2006). As will be discussed later in *Controllability and predictability of stress*, a sense of agency and control over the environment is thought to have a beneficial effect for a range of taxonomic groups. Although SIT is not appropriate here, certain coping skills involved in the treatment may be beneficial, for example problem-solving skills.

#### Inoculation from major lifetime stressors

Alongside daily stressors related to life in captivity, all orangutans in rehabilitation will have experienced at least one major life stressor, i.e. capture and travelling to the rehabilitation centre. However, depending on the prior history of each orangutan, they are likely to have experienced additional major stressors leading up to their rescue, such as witnessing the death of their mother, as well as stressors during their time in rehabilitation, for example veterinary interventions. Although some studies identified a positive linear relationship between cumulative lifetime adversity and the odds of being diagnosed with a stress-related disorder (Gerber *et al.* 2018; Fernandez *et al.* 2020), there is more robust evidence for a U-shaped relationship. In a large study involving a stratified random-digit-dial telephone survey with 2,398 members of the public, Seery *et al.* (2010) found that individuals with some experience of lifetime adversity had lower ratings for distress, functional impairment, and PTSD symptoms, higher ratings for life satisfaction, and were least affected by recent adversity than those with no experience of prior adversity, or those with high exposure to adversity (categorised as mean + 1 SD; ~ 87th percentile). These results are supported by Seery *et al.* (2013), who reported a similar U-shaped relationship between lifetime adversity and pain-induced catastrophising and negative affect, with moderate numbers of prior major life stressors (two to seven) associated with the highest resilience to stress. If a U-shaped relationship between life stressors and resilience exists for orangutans, it is very important that an ‘upper limit’ of life stressors is established, so it can be avoided. However, because orangutans’ life histories prior to arrival at the rehabilitation centres are often unknown, this will be difficult to achieve. The concept of stress inoculation and the effects of prior adversity will be discussed further in the *Animal intervention* section of this paper.

## Section two: Animal resilience treatments

In this section, stress and resilience treatments for animals are broadly separated into five categories: direct stress procedures designed to elicit pain/fear/discomfort; separation from the group/mother; environmental deprivation/enrichment; social stress, including exposure to strangers or dominant individuals; and exercise regimes. These are different to the human resilience categories shown in Table 1 as the five categories described here relate to forms of treatment, rather than themes of resilience within different types of talking therapies. There were one to four treatments per study, with a mean of 1.36 treatments and the great majority of papers using a single experimental treatment, alongside a control (where applicable) (201/266). Many studies, particularly those involving rodents, used well-established behavioural tests to measure aspects of resilience and stress indicators, summarised in S3 Appendix (Supplementary material). A comprehensive overview of animal resilience treatments is shown in Tables 2(a)–(e). Since exactly the same event, with exactly the same effect on the animal, could either be an intended stress (i.e. stress-related resilience treatments) or an unintended ‘accidental’ stress (e.g. unavoidable/unpredictable stressors), treatments have been grouped by the form of stressor, rather than by whether the stress constituted a deliberate treatment or not, or the direction of the effect (i.e. whether positive or negative). The effect of each stressor on resilience must first be established before decisions are made about whether these can be turned into intentional treatments. It is also important to be able to compare the difference in effect of similar kinds of stress side-by-side. For example, short separations from the group/mother are generally considered helpful, whereas prolonged separations are generally deemed unhelpful to resilience. Therefore, Tables 2(a)–(e) and the remainder of this section groups treatments by their form of stress, in descending order of the number of relevant papers.

**Table:** Key to Tables 2(a)–(e)

Strength of evidence (E)(The number and proportion of studies which support, i.e. provide evidence for, the effect on resilience category - see ‘effect on resilience’ key, below)				
Weak evidence	Limited evidence	Inconclusive	Some evidence	Strong evidence
*Single study*	*<5 studies*	*5+ studies,* *40-59% support effect*	*5+ studies,* *60-79% support effect*	*5+ studies,* *80-100% support effect*
				

Effect on resilience (R)				
Negative	Neutral-Negative	Neutral/unclear	Neutral-positive	Positive
*>70% studies have negative effect*	*>70% studies have negative and/or no effect*	*Either no studies with a clear positive/negative effect, or equal numbers of studies with conflicting effects*	*>70% studies have positive and/or no effect*	*>70% studies have positive effect*
				

### Generic stressors/stress schedules

It is important to understand the effects of direct stress procedures designed to elicit pain, fear, or discomfort, not only to investigate how prior stress influences resilience, but also whether milder forms of stress can have an inoculating effect. There is strong evidence that acute (single stress session) and chronic (over a period of at least ten days) stressors (see S1 Appendix; Supplementary material) cause a range of long-lasting negative effects in rodents, including increased anxiety and depression, heightened fear expression during re-exposure to stressors, and reduced cognitive and behavioural flexibility (see Table 2[a]). However, the evidence for the effects of restraint stress, or short periods of stress (3–7 days) of various kinds, such as electric shock, predator odour, forced swim, and/or elevated platform stress (see S1 Appendix; Supplementary material), on rodents is less clear-cut. There is contradictory evidence, with some studies showing positive outcomes, and others showing negative outcomes. As with separation stress, companionship with a conspecific may protect against the negative effects of direct stress. For example, one study of the effect of chronic unpredictable stress on male mice found that 30-min periods spent with a female mouse in between stressors ameliorated some depressive effects of the stress (Li *et al.* 2022). Another study found that stimulation of rats by humans, i.e. stroking them at a rate of 5 cm s^–1^ for ten minutes prior to chronic unpredictable mild stress, helped to protect against increased anxiety and depression in subsequent behavioural tests (Walker *et al.* 2022).

**Table 2(a).** Summary of generic stressor/stress treatment effects by taxonomic group, and sex

Treatment	Taxonomic group	S	E	R	Notes on treatment	Most relevant studies
Acute stress (see S1 Appendix) *(single acute stress session)*	Rodents	B			Strong evidence that ‘single prolonged stress’ and other acute stressors can cause a range of negative effects, including heightened fear expression during re-exposure to stressors, increased anxiety and depression, and reduced behavioural flexibility.	(Adamec & Shallow 1993, Tsoory *et al.* 2007, Bazak *et al.* 2009, Eagle *et al.* 2013, Perrine *et al.* 2016, Le Dorze & Gisquet-Verrier 2016, Liu *et al.* 2017, Chen *et al.* 2018, Denny *et al.* 2021, Gonzalez *et al.* 2021, Nahvi *et al.* 2023)
		F			Conflicting studies, unclear effect on female rodents.	
	Dogs	?			Two studies intentionally induced learned helplessness (see S1 Appendix) in dogs (*Canis familiaris*) in an electric shock session.	(Overmier & Seligman 1967, Seligman *et al.* 1968)
Short periods of stress (e.g. electric shock, predator odour, forced swim, or elevated platform stress) (see S1 Appendix) *(3-7 days)*	Rodents	M			Contradictory evidence of effects, mostly negative, and possibly age-dependent.	(Avital *et al.* 2006, Tsoory & Richter-Levin 2006, Toledo-Rodriguez & Sandi 2007, Peleg-Raibstein & Feldon 2011, Wilkin *et al.* 2012, Baugher & Sachs 2022)
		F			Contradictory evidence of positive and negative effects, possibly age-dependent. One study found reduced freezing in response to a fear cue in adolescent females, but not males.	
	Birds	F			One study of Japanese quail (*Coturnix japonica*) found that one week of unpredictable stress improved spatial learning and behavioural flexibility.	(Calandreau *et al.* 2011)
Chronic stress (see S1 Appendix) *(10-48 days)*	Rodents	B			Strong evidence of chronic stress causing a range of negative effects. Contradictory evidence regarding importance of age and sex. Mixed evidence of the effect of mild/predictable chronic stress, with some studies showing benefits and others showing increased anxiety and depression	(Katz *et al.* 1981, Garcia-Marquez & Armario 1987, Ducottet & Belzung 2004, Mineur *et al.* 2007, Pohl *et al.* 2007, Swiergiel *et al.* 2007, Bondi et al. 2008, Toth *et al.* 2008, Bourke & Neigh 2011, Muhammad & Kolb 2011, Castro *et al.* 2012, Chaby *et al.* 2013, 2015a, 2015b, Remus *et al.* 2013, Burgdorf *et al.* 2017, Yohn & Blendy 2017, Cotella *et al.* 2019, Li *et al.* 2022, Lyte *et al.* 2022, Strekalova *et al.* 2022, Walker *et al.* 2022)
Restraint stress *(usually 6 hours daily from 10 days to 4 weeks)*	Rodents	M			Contradictory results, with some studies showing negative effects. However, some forms of restraint stress, particularly shorter periods of restraint (i.e. 5 minutes to 1 hour), supporting positive effects of restraint stress; for example, reduced anxiety and depression, and increased exploration, spatial memory, and novel object recognition.	(Luine *et al.* 1994, 1996, Bowman *et al.* 2001a, Beck & Luine 2002, Luine 2002, Wood *et al.* 2008, Doremus-Fitzwater *et al.* 2009, Parihar *et al.* 2011, Hoffman *et al.* 2011, Chiba *et al.* 2012, Eiland & McEwen 2012, Shi *et al.* 2021, Torrisi *et al.* 2021, Lyte *et al.* 2022, Yan *et al.* 2023, Campos-Cardoso *et al.* 2023)
		F			Most studies had negative effects, but one study found a positive effect of restraint stress on spatial memory in females only, particularly those in proestrus, compared to controls.	
	Agricultural	F			One sheep (*Ovis aries*) study showed that three days of 6h restraint sessions led to a more optimistic judgement in a positive/negative bias test than unrestrained controls.	(Doyle *et al.* 2010)
Controllable/ predictable stress	Rodents	M			Most studies showed positive effects of being able to control stress, in protecting against stress and depression when exposed to later stressors, as well as enhancing social learning and the ability to discriminate between positively and negatively reinforced odours. Some of these benefits emerged over time.	(Prince & Anisman 1984, Brown *et al.* 2001, Christianson *et al.* 2008, Amat *et al.* 2010, Kubala *et al.* 2012, Suo *et al.* 2013, Lucas *et al.* 2014, Baratta *et al.* 2018)
		F			Only one study, which did not show an effect of being able to control stress.	
	Agricultural	F			One study found a positive effect of being able to control stress in sheep (*Ovis aries*), another study found no effect of stress predictability.	(Greiveldinger *et al.* 2007, 2009)
	Dogs	?			Two dog (*Canis familiaris*) studies induced learned helplessness through loss of controllability. In one study, this was later reversed by physically forcing the dogs to escape the stressor.	(Overmier & Seligman 1967, Seligman *et al.* 1968)
	Birds	F			One chicken (*Gallus domesticus*) study found that loss of both controllability and predictability increased frustration.	(Zimmerman & Koene 1998)

(Grey-shaded cells highlight primate studies; S = sex (F = female, M = male, B = both/mixed, ? = not stated)

However, some taxonomic groups may experience benefits from stress treatments. For example, one week of unpredictable stress in Japanese quail (*Coturnix japonica*) led to increased spatial learning and behavioural flexibility when tested the next day (Calandreau *et al.* 2011). One sheep (*Ovis aries*) study found that three days of 6-h restraint sessions led to more optimistic judgements than unrestrained controls in a positive/negative bias test (Doyle *et al.* 2010). However, as the subjects were not later retested, the duration of these benefits is unclear. Behavioural flexibility, cognitive skills, and optimism are all thought to be aspects of resilience (Masten *et al.* 1990; Mandleco & Peery 2000), and therefore may help individuals in overcoming future stress (see *Human resilience*).

Some rodent studies indicate that certain characteristics are conducive to resilience against stress, for example low anxiety and exploration, low emotionality, and positive affect (see S1 Appendix; Supplementary material) (Ducottet & Belzung 2004; Mällo *et al.* 2009; Castro *et al.* 2012). It is interesting that Castro *et al.* (2012) found that a combination of low anxiety and low exploration led to resilience against stress in rats, as this contradicts the fact that curiosity, physical activity, and self-motivation are all thought to be important to resilience. In addition, once released into the wild, orangutans will need to exhibit enough targeted exploratory behaviours appropriate to specific situations to be able to locate food and establish a home range without unnecessary exposure to risk. It is possible that the importance of low anxiety and exploration is specific to resilience in rats, or the laboratory environment, and so further research needs to be carried out here to understand the relationship between individual characteristics and levels of resilience. Liu *et al.* (2017) identified a resilient group of subjects who showed earlier signs of anxiety and depression following acute stress, but had no behavioural deficits later in life. In another rat study, rough-and-tumble play during chronic unpredictable stress protected against any negative effects (Burgdorf *et al.* 2017). These protective characteristics should be further explored, to see whether they are relevant in other contexts.

#### Controllability and predictability of stress

Uncontrollable stress can lead to ‘learned helplessness’ (see S1 Appendix; Supplementary material), a term which describes the failure to respond to avoidable shocks, due to prior experience of inescapable traumatic shock (Overmier & Seligman 1967). This should be taken seriously in the context of orangutan rehabilitation, where space is limited and orangutans have very little control or ability to make decisions to change their situation. For example, they are restricted in what and when they eat, who they have social contact with, and where they can go while in the rehabilitation centre. Maier (2001) found that inescapable stress followed by repeated exposure to the specific context in which stress took place prolonged depression and learned helplessness in rats indefinitely. Captive orangutans may be repeatedly exposed to stressful contexts such as relatively small enclosures or visits from the vet. Repeatedly ‘refreshing’ the stress associated with captivity and small environments may have long-lasting detrimental effects. It would be beneficial to identify coping strategies currently used by orangutans in rehabilitation to manage long-term stressors.

There is strong evidence that there are positive effects of being able to control electric shock stress in male rodents, in protecting against stress and depression when exposed to a variety of future stressors and enhancing social exploration and the ability to learn how to discriminate between positively and negatively reinforced odours (see Table 2[a]). Similar benefits are seen in sheep. Greiveldinger *et al.* (2009) found that teaching lambs how to control an airblast to the muzzle during feeding led to lower emotionality and vigilance compared with subjects who did not have the opportunity to control the stressor. Predictability can also reduce the impact of stress. Lambs that experienced a predictable sudden event – either due to regularity of appearance, or because it was associated with a cue – had a reduced startle response and less disruption to feeding than those that experienced an unpredictable event (Greiveldinger *et al.* 2007). Although neither predictability nor controllability of the stressor had a noticeable effect on chicken (*Gallus domesticus*) behaviour, loss of both had the effect of increasing their frustration, measured by higher levels of vocalisations (Zimmerman & Koene 1998). When learned helplessness has occurred, two studies on dogs and rats, respectively, found that it can be reversed by force, i.e. by a human physically dragging the animal away from the source of electric shock (Seligman *et al.* 1968, 1975). Although the ethical problems with these methods are substantial, it does indicate that recovery from learned helplessness is theoretically possible.

#### Direct stress summary

This section clearly demonstrates the potential harms from both acute and chronic forms of stress. However, some forms of milder, less extended, and more controllable forms of stress may have potential benefits. It is natural for orangutans to experience stressful situations in the wild, and it is therefore important for rehabilitant orangutans to be given the opportunity to develop resilience by successfully overcoming a range of different challenges prior to release. Puzzle-boxes, or treatments such as a controllable airblast during feeding, may be worth considering, as they provide a mildly frustrating – but ultimately rewarding – challenge that can be resolved through persistence and problem-solving. The importance of effort-based reward is something that has been addressed in human studies. For example, Dweck (1975) found a way to ‘treat’ learned helplessness in human children, by teaching them to take responsibility by attributing failures to a lack of effort, rather than ability. Appropriate difficulty levels of cognitive enrichment will be discussed further in *Environmental conditions* below. There may also be specific personality traits that help to protect against stress, such as low anxiety, low emotionality, and positive affect, which may help orangutans adapt to a new environment. However, although generally low levels of anxiety may be beneficial, it is important that orangutans are still responsive to genuine threats, so that these can be avoided. Therefore, interventions that induce an anxious response to specific threats, for example snakes and humans, can still be utilised, even if overall low anxiety levels are desirable for resilience in orangutans.

### Separation

This section is particularly relevant for three reasons. The first is that nearly all orangutans in rehabilitation have been separated from their mothers at an early age, which is likely to cause considerable distress and persisting trauma considering the long-lasting mother/infant bond in orangutans (van Noordwijk & van Schaik 2005). The second reason is that human caretakers will not have the same intensity and duration of contact that the orangutans would normally have with their mother. On the other hand, too much contact with humans may increase dependence on human care (unpublished OVAG questionnaire, L Bridgeland-Stephens 2020) and lead to orangutans seeking out humans after they have been released into the wild, which could pose a considerable risk to the orangutans. The balance between ‘tough’ and ‘motherly’ approaches to caretaking is discussed in more detail by Palmer (2020). Therefore, it is important to understand the effects of the presence and/or absence of a caretaker, so that an optimal amount and appropriate form of contact between humans and orangutans in a rehabilitation setting can be determined. Lastly, understanding the effects of separation has implications when considering the logistics of housing orangutans in groups, and the potential effects of isolation if this is not possible.

#### Brief separations

Brief daily separation treatments (15–60 min) have a range of beneficial effects, and this is supported by a strong body of evidence. Over the past two decades, a series of stress inoculation studies were carried out on squirrel monkeys (*Samiri sciureus*) raised in a normal, species-typical way in a laboratory environment. These involved moving subjects from their natal group to an enclosure adjacent to an unfamiliar group for one hour each week, for a period of ten weeks during adolescence, with the purpose of inoculating the monkeys against future stress. This treatment resulted in benefits such as reduced anxiety, increased time spent exploring and interacting with novel objects, and enhanced inhibitory control (Parker *et al.* 2004, 2005, 2007, 2012, 2019). However, it should be noted that these studies were all carried out by the same research team, and some research subjects were used for multiple studies, so caution should be taken in generalising the results to other populations. Many rodent studies found positive effects in adulthood of 15 min of daily keeper handling (involving separation from the litter) during infancy or adolescence, including protection against stress, a reduction in anxiety, depression, and fear, and increased playfulness and exploration (see Table 2[b]). These brief separation treatments may be transferable to the context of orangutan rehabilitation. Exposing young infant orangutans to mild separation stress by removing them from their social group and placing them next to unfamiliar orangutans may have stress-inoculating benefits. However, careful management decisions would need to be made on an individual basis regarding the appropriateness of this method. In rehabilitation centres, detailed knowledge of prior traumatic events experienced by individual orangutans is usually not available. As the studies detailed above are carried out on normally reared laboratory animals with known histories, there is less risk of unexpected trauma surfacing during the separations. This potential interaction between prior stress and the effect of separation is related more generally to the nature of the relationship between life stressors and resilience, discussed in the section above on human resilience.

**Table 2(b).** Summary of maternal/group separation treatment effects by taxonomic group, and sex (96/266 papers reviewed)

Treatment	Taxonomic group	S	E	R	Notes on treatment	Most relevant studies
Brief separations *(15 minutes to 1 hour)*	**Primates**	B			Moving Squirrel monkeys (*Samiri sciureus*) next to an unfamiliar group for one hour weekly for ten weeks during adolescence led to reduced anxiety, increased exploration and interaction with novel objects, and enhanced inhibitory control.	(Parker *et al.* 2004, 2005, 2012, 2019, Lyons & Parker 2007)
	Rodents	B			In some studies, 15 minute daily maternal separation and handling had no effect compared to controls. Where an effect was seen, brief separations tended to protect against stress, reduce emotionality, anxiety, depression, and fear, and increase playfulness, exploration, and vigilance.	(Núñez *et al.* 1995, 1996, McIntosh *et al.* 1999, Caldji *et al.* 2000, Siviy & Harrison 2008, Stamatakis *et al.* 2008, Mrdalj *et al.* 2016)
Longer separations *(2-4.5 hours daily for 1-3 weeks)*	**Primates**	B			Marmosets (*Callithrix jacchus*) experienced a range of negative effects after 26 days of 30–120 minute daily separations, for example reduced levels of activity, play, social contact, motivation, and increased impulsivity.	(Pryce *et al.* 2004)
	Rodents	M			Longer separations generally led to a range of negative effects, including increased anxiety, fear, emotional reactivity, and depression, cognitive impairments, and reduced exploration. However, one study showed that predictable maternal separations can reduce anxiety resulting from stress (Shi *et al.* 2021).	(Ogawa *et al.* 1994, Caldji *et al.* 2000, Penke *et al.* 2001, Bowman *et al.* 2001b, Kalinichev *et al.* 2002, Romeo *et al.* 2003, Daniels *et al.* 2004, Gardner *et al.* 2005, Uysal *et al.* 2005, Colorado *et al.* 2006, Aisa *et al.* 2007, 2008, Lee *et al.* 2007, Marais *et al.* 2008, Mourlon *et al.* 2010, Hulshof *et al.* 2011, Leussis *et al.* 2012, Baudin *et al.* 2012, Eiland & McEwen 2012, Marco *et al.* 2013, Sun *et al.* 2014, Zalosnik *et al.* 2014, Shu *et al.* 2015, Bian *et al.* 2015, Farrell *et al.* 2016, Grassi-Oliveira *et al.* 2016, Wei *et al.* 2018, Shi *et al.* 2021, Baugher & Sachs 2022)
		F			Most studies show a negative effect, or no effect, but a small number of mixed-sex studies show a sex-specific positive effect in females, in the form of decreased fearfulness and/or anxiety. One study identified these benefits in females in diestrus only (Romeo *et al.* 2003).	
Prolonged isolation *(> 24 hours)* or surrogacy	**Primates**	B			Infant macaque species (*Macaca mulatta/fascicularis*) in laboratory environments were negatively affected by prolonged separation or surrogacy (either peer, or inanimate wire/cloth ‘surrogate’). Negative effects included increased stereotypies, reduced activity and exploration, and abnormal social interactions.	(Suomi *et al.* 1970, Sackett 1972, Suomi & Harlow 1972, Novak & Harlow 1975, Röder *et al.* 1989, Feng *et al.* 2011, Corcoran *et al.* 2012)
	Rodents	M			Mixed results, with most studies showing no effect or a negative effect, and a small number of studies showing a positive effect.	(Grippo *et al.* 2007, 2008, Abraham & Gruss 2010, Weintraub *et al.* 2010, Han *et al.* 2011, Hong *et al.* 2012)
		F			Mixed results, with most studies showing no effect, and equal numbers of studies showing positive and negative effects.	

(Grey-shaded cells highlight primate studies; S = sex (F = female, M = male, B = both/mixed, ? = not stated)

#### Longer separations

There is fairly consistent evidence in rodents that longer periods of separation (2–4 h daily) have a range of negative effects (see Table 2[b]), and this is also supported by a primate study (Pryce *et al.* 2004). Wild orangutan infants are in near-continuous bodily contact with their mother for the first two years of life, only gaining full locomotory independence at 5–6, and sharing her nest until weaning at 6–8 years old (van Noordwijk & van Schaik 2005; van Adrichem *et al.* 2006). Even years after gaining independence, orangutans have been observed to occasionally ‘visit’ their mother at eleven years old (van Noordwijk *et al.* 2009). However, early maternal separation is inevitable for nearly all orangutans rescued from the illegal wildlife trade, as the process of capture usually requires the death of the infants’ mother (Nijman 2005). This means that infant orangutans rely mostly on a combination of human caretakers and peer-rearing. A study of laboratory chimpanzees (*Pan troglodytes*) found that individuals who had been separated from their mothers earlier, and isolated for longer, were less social, less dominant, and more affected by stressful experiences (Reimers *et al.* 2007). Compared with maternally raised individuals, same-species surrogacy leads to a reduction in locomotion and time spent exploring in a number of other studies (Sackett 1972; Röder *et al.* 1989; Corcoran *et al.* 2012). In the context of orangutan rehabilitation, early maternal separation will unavoidably have a detrimental impact. However, social support may help to ameliorate stress; in one marmoset study (*Callithrix kuhli*), anxiety experienced upon moving to a new enclosure was reduced by the presence of their breeding partner during the transition (Smith *et al.* 1998).

#### Prolonged isolation

Orangutans in the illegal wildlife trade may spend prolonged periods of time, even years, with no conspecific contact. However, it may be possible to reverse certain behavioural effects of isolation. Some 1970s primate studies measured the consequences of extreme forms of separation in rhesus macaques. Although disturbing in their methods, two studies have demonstrated successful reversals of the traumatic effects of prolonged isolation. Suomi and Harlow (1972) managed to reverse the effects of six months of isolation in four male rhesus monkeys, by socially exposing them to younger, normally reared female monkeys. After six months of ‘social rehabilitation’, isolated monkeys demonstrated similar social behaviour to controls and an absence of stereotypies. Similar results were achieved in another study of the same species after an entire year of total isolation (Novak & Harlow 1975). Particularly traumatised orangutans may therefore benefit from peer support in carefully arranged group housing, so that individuals who are coping more effectively can experience a positive effect by association (see *Social stress* for potential risks). Another technique which has been borrowed from the rehabilitation of ex-laboratory primates is increasing the ratio of caretakers to orangutans in order to monitor progress more carefully, although this may result in excess humanisation (Palmer 2020).

#### Separation stress summary

There are many potential harms from extended periods of maternal/group separation. However, briefer separations could be used as a mechanism to create a positive, stress-inoculating effect, as seen in the squirrel monkey studies by Parker and colleagues. Maintaining contact with a social peer during separations may help to reduce associated stress. These kinds of ‘gentle’, sporadic separations should be explored as a potentially appropriate treatment for orangutans in rehabilitation, applying a principle of caution and initially trialling with very short periods of time.

### Environmental conditions

Despite best efforts, captive environments can never equal the spatial and temporal complexity of life in the forest, and the level of enrichment provided by wild environments. However, there is great potential here, as environmental conditions can be relatively easily controlled in a rehabilitation setting. The treatments in this section either involve environmental enrichment or environmental stress/deprivation. Understanding the negative effects of environmental deprivation is important for orangutan rehabilitation, as most individuals will experience poor conditions before arriving at the rehabilitation centre. Even during rehabilitation, captive environments are relatively deprived of enrichment compared to the complex and dynamic forest environment, and so it is important that opportunities for enrichment are maximised within these constraints.

#### Environmental enrichment

Environmental enrichment can have a variety of benefits in rodents, including protecting against the negative effects of future stress on anxiety, depression, fear, and emotional reactivity, as well as increasing activity levels, learning ability, and spatial memory (see Table 2[c]). Cognitive challenges can also have beneficial effects, with effort-based reward training in rats leading to improved persistence and problem-solving (Bardi *et al.* 2012; Lambert *et al.* 2014). This makes sense in terms of having the opportunity to exercise agency (see S1 Appendix; Supplementary material), and overcoming a certain degree of challenge having the effect of eliciting a positive emotional state (Clark 2011). There is also strong evidence that environmental enrichment improves the well-being of pigs (*Sus scrofa domesticus*), including promoting long-term memory, mobility, and diversity of behaviour, and reducing emotionality (De Jong *et al.* 2000; Wemelsfelder *et al.* 2000; Puppe *et al.* 2007). However, the form of enrichment used may be important. In a study comparing the effects of natural and artificial (e.g. plastic/manufactured) novel object enrichment on rats, Lambert *et al.* (2016) observed that rats interacted nearly three times more with natural enrichment items than artificial enrichment items matched according to their functionality and purpose (i.e. climbing, shelter, or manipulation), as well as having a greater reduction in anxiety-like behaviours. Although natural enrichment may be preferable, the practicalities of this with larger animals can be complicated, as enrichment and enclosure features must also be durable and not easily destroyed. Chappell and Thorpe (2022) argue that non-natural enclosure modifications can simulate the mechanics of a natural environment and provide similar physical and cognitive challenges to those provided by wild environments.

**Table 2(c).** Summary of environmental conditions treatment effects by taxonomic group, and sex (41/266 papers reviewed)

Treatment	Taxonomic group	S	E	R	Notes on treatment	Most relevant studies
Env. Stress/ deprivation *(for rodent studies, ‘early life stress’ consists of 1 week of wire floors and restricted bedding)*	**Primates**	B			One bonnet macaque (*Macaca radiata*) study found that variable foraging demand (i.e. periodically having to search for food under woodchip) had a negative effect on infant exploration, as infants were less likely to break contact with their mother to explore a novel environment.	(Andrews & Rosenblum 1993)
	Rodents	M			Most studies show a negative effect of early life stress, for example increased anxiety, impaired spatial memory and novel object recognition, and reduced exploration. Some (limited) evidence to suggest that negative effects are more prolonged in males than females.	(Brunson *et al.* 2005, Dalle Molle *et al.* 2012, Machado *et al.* 2013, Bath *et al.* 2017, Goodwill *et al.* 2019)
		F			Most studies show a negative effect of early life stress, for example increased anxiety in an open space. Some evidence of a sex-specific depressive effect of early life stress on females. Possible improved recovery from early life stress in females compared to males.	
	Birds	B			Two zebra finch (*Taeniopygia guttata*) studies support some benefits of low food conditions, including faster spatial learning and exploration. A small study (6 individuals) of starlings (*Sturnus vulgaris*) found a detrimental effect of environmental enrichment followed by deprivation.	(Bateson & Matheson 2007, Krause *et al.* 2009, Kriengwatana *et al.* 2015)
	Fish	B			One Panamanian bishop (*Brachyrhaphis episcopi*) study found that high-risk environments were associated with increased activity and exploration, both of which are indicators of resilience%	(Archard & Braithwaite 2011)
Env. Enrichment	Rodents	B			There is a strong body of evidence that environmental enrichment can protect against stress, anxiety, depression, fear, and emotional reactivity, as well as increasing activity levels, learning ability, and spatial memory. Some of these effects may be dependent on age, severity of stress, and type of enrichment. However, these benefits may be lost when enriched conditions are reverted back to baseline conditions.	(Schrijver *et al.* 2002, Francis *et al.* 2002, Benaroya-Milshtein *et al.* 2004, Leggio *et al.* 2005, Cui *et al.* 2006, Zambrana *et al.* 2007, Hattori *et al.* 2007, Ilin & Richter-Levin 2009, Harati *et al.* 2013, Lambert *et al.* 2016, Sampedro-Piquero *et al.* 2016, Strzelewicz *et al.* 2019, Crawford *et al.* 2020, Luo *et al.* 2022, Kent *et al.* 2022)
	Agricultural	B			Strong evidence for a positive effect of environmental enrichment on pigs (*Sus domesticus*), including greater mobility, diversity of behaviours, and reduced emotionality. However, enrichment followed by deprivation had a negative effect, for example increasing pessimism in a cognitive bias test.	(De Jong *et al.* 2000, Wemelsfelder *et al.* 2000, Bolhuis *et al.* 2006, Puppe *et al.* 2007, Douglas *et al.* 2012)

(Grey-shaded cells highlight primate studies; S = sex (F = female, M = male, B = both/mixed, ? = not stated)

However, moving from an enriched to a barren environment can be more harmful than only experiencing barren environments (Bolhuis *et al.* 2006). In studies of pigs, moving from an enriched to a barren environment has been found to reduce activity levels (Bolhuis *et al.* 2006), and increase pessimism in a cognitive bias test used to measure attitudes towards an ambiguous cue (Douglas *et al.* 2012) Similarly, starlings (*Sturnus vulgaris*) who had moved from enriched to barren environments had increased pessimism in a cognitive bias test (Bateson & Matheson 2007). This has implications for orangutan rehabilitation, as orangutans are likely to experience a fluctuation of environmental conditions, from wild environments to extremely poor captive conditions (for illegally traded individuals), to improved conditions at the rehabilitation centre with varying levels of enrichment, depending on the stage of rehabilitation. It is not clear from the literature how a series of multiple changes in condition would affect individuals, and this makes it very difficult to be sure how changing environmental conditions are likely to impact orangutans in rehabilitation. However, it seems likely that an increasing trajectory of environmental complexity would be beneficial, with orangutans in rehabilitation experiencing gradual increases in enrichment and autonomy and, as far as possible, avoiding a reversal in conditions.

#### Environmental stress/deprivation

Most evidence suggests that environmental deprivation, for example wire floors and restricted bedding during infancy, has a range of negative effects in rodents, for example increased anxiety, impaired spatial memory and novel object recognition, and reduced exploration (see Table 2[c]). However, the studies on environmental stress in birds and fish had less straightforward results. In zebra finches (*Taeniopygia guttata*), a low quality diet led to faster spatial learning and exploration compared to individuals on a high quality diet (Krause *et al.* 2009; Kriengwatana *et al.* 2015), although spatial memory was impaired (Kriengwatana *et al.* 2015). In the Panamanian bishop (*Brachyrhaphis episcopi*), high-predation environments were associated with increased activity and exploration (Archard & Braithwaite 2011). Exposure to environmental stressors such as food availability, or risk of predation, may therefore have some potential benefits in fish and birds, such as increased motivation and exploration. However, a study of foraging demand on bonnet macaques (*Macaca radiata*) found – perhaps surprisingly – that infants whose mothers had to periodically forage in woodchip were less likely to explore a novel environment than those for whom food was easily available (Andrews & Rosenblum 1993). This indicates that there may be taxonomic and context-specific differences in how various types of environmental stress are experienced, and that human-controlled environmental deprivation may have different effects to more naturally arising situations such as competition for food.

#### Environmental conditions summary

The damaging effects of early life stress are extremely relevant to the context of orangutan rehabilitation, as many infant orangutans are rescued from the illegal wildlife trade and found in deprived conditions. In situations where an orangutan is rescued from a human environment, it is reasonable to assume that some detrimental effects of early life stress, e.g. increased anxiety and reduced exploration, may carry through into adulthood, emerging over time. Environmental enrichment can protect against stress, reduce anxiety, depression, and fear, and promote physical activity and spatial memory. However, moving animals from enriched to unenriched environments may have unintended negative effects. This shift in environmental conditions can occur in several circumstances. For example, despite existing enrichment provision in rehabilitation centres, e.g. hammocks and feeding enrichment (Damerius *et al.* 2017), orangutans who have been living in the wild before arriving at a rehabilitation centre will experience a large shift in conditions, from a complex forest environment to the relative deprivation of captivity. However, even within rehabilitation centres there is limited space, and orangutans may need to be temporarily housed in smaller enclosures than those to which they are accustomed. For example, infant orangutans too old for ‘forest school’, i.e. learning skills under human supervision in an area of open forest during the day, may need to be housed for some years in an enclosure before they are old enough to be released into the wild (R Jaya, personal communication 2022). Similarly, orangutans who spend time on a ‘pre-release island’ to experience semi-independent living may be temporarily returned to their enclosure before being released into the wild. In these situations, the regression back to an enclosed environment may have considerably negative effects, including increasing pessimism, which is discussed in the following section. This could be heightened if enclosed environments have negative associations, for example in orangutans who have experienced life in the illegal wildlife trade or who have other prior experience of enclosed environments. Regression to a smaller environment is also likely to reduce levels of physical activity, which in turn is likely to have a negative impact on resilience.

In humans, optimism and pessimism are considered to influence the capacity for resilience; for example, affecting self-perception, environmental perception, and how information is processed and actioned (Forgeard & Seligman 2012). Hobfoll (2002) argues that optimism, self-esteem, and a sense of agency, overlap and tend to be correlated. Optimists can actively approach challenges in a constructive way, perceiving the possibility to act in order to alter outcomes (Forgeard & Seligman 2012). Therefore, an optimistic outlook may help orangutans maintain a sense of control over their surroundings. However, it could also be argued that temporary realistic pessimism may be more appropriate in the face of danger, to prepare for worst-case scenarios and lower expectations of success to prevent disappointment (Forgeard & Seligman 2012). Therefore, a careful balance should be maintained here; the beneficial effects of enrichment can be used in parallel with some constructive environmental challenges that encourage persistence and problem-solving. This requires an understanding of appropriate difficulty levels, so that the challenge presents some degree of frustration, but ultimately elicits a positive emotional state rather than apathy, boredom, or a negative emotional state (Clark 2011). In addition, long-term planning is important to ensure that the positive effects are long-lasting and maintained up until the point orangutans are released. It would also be helpful to establish an understanding of current enrichment practices in rehabilitation centres, to measure the effectiveness of different methods and explore the relationship between environmental enrichment and optimism/pessimism in orangutans.

### Social stress

As orangutans are semi-solitary animals who live in dispersed societies, only forming temporary aggregations (Galdikas 1985; Malone *et al.* 2012; Roth *et al.* 2020), proximity with large numbers of other orangutans in rehabilitation centres may cause stress, particularly where direct conflict between conspecifics occurs. However, the sociability of orangutans may be partially dependent upon resource availability. For example, Schuppli *et al.* (2017) found that Sumatran orangutans (*Pongo abelii*) in an area with higher food availability were more sociable and, in turn, more exploratory, than Bornean orangutans (*Pongo pygmaeus warmbii*) in an area with lower food availability. This indicates that there is some degree of behavioural flexibility, and/or species differences, in the sociability of orangutans. Therefore, appropriate levels of sociability in orangutan rehabilitation centres may vary depending on a number of factors, including localised differences and individual tendencies.

While in captivity, and after being released into the wild, it is important for orangutans to be able to communicate appropriately and navigate social situations in an effective way. Among treatments reviewed, the most common type of social stress included brief (often 15 min) exposure to a larger, more dominant, strain of rat, with the interaction ending in ‘social defeat’ (see S1 Appendix; Supplementary material), i.e. forced subordination, for the focal animal. Another treatment, called ‘chronic social stress’ or ‘social instability stress’, involved rotating individuals around unfamiliar conspecifics over consecutive days. Both forms of stress were found to have a negative effect in rodents (see Table 2[d]). As with other forms of stress, the negative effects of social defeat stress in male rodents can be ameliorated with regular female companionship throughout the period of stress (Shi *et al.* 2023). There is also evidence for social stress being contagious, and observing a conspecific experiencing social defeat can lead to depressive and anxious behaviours, and memory deficits in rats (Patki *et al.* 2014), although the close social structure in rats may heighten the negative effect of this treatment. However, if applicable to orangutans, this aspect of social stress would be particularly relevant in the context of great ape rehabilitation, as individuals are likely to be exposed to other stressed individuals, potentially exacerbating their own stress, learning to copy stress-related behaviours such as stereotypies, and/or complicating or prolonging the rehabilitation process. Conversely, indirect exposure to a dominant individual can have a stress-inoculating effect in rodents. This treatment does not involve social defeat, but rather confinement to a small part of an enclosure which houses a dominant individual. This can lead to a range of positive effects, including a more active coping strategy, increased exploration and social interaction, and reduced anxiety (Brockhurst *et al.* 2015; Lyons *et al.* 2018; Ayash *et al.* 2020).

**Table 2(d).** Summary of social stress treatment effects by taxonomic group, and sex (26/266 papers reviewed)

Treatment	Taxonomic group	S	E	R	Notes on treatment	Most relevant studies
Social defeat stress (see S1 Appendix)	Rodents	M			Strong evidence to support a negative effect of social defeat stress, even if the subject is only witnessing a conspecific experiencing social defeat, and the defeat is not experienced directly. These negative effects include increased anxiety, impulsivity, and depression, impaired social interactions, and memory deficits.	(Wommack *et al.* 2004, Jacobson-Pick *et al.* 2011, Patki *et al.* 2014, Santarelli *et al.* 2017, Bravo-Tobar *et al.* 2021, Jing *et al.* 2021, Lee *et al.* 2021, Li *et al.* 2021, Lu *et al.* 2021, Calpe-López *et al.* 2022, Wang *et al.* 2022, Willmore *et al.* 2022, Shi *et al.* 2023)
		F			One study showing increased anxiety in an open field test, particularly in the metoestrus and dioestrus phase, but no effects in other behavioural tests of anxiety and depression.	(van Doeselaar *et al.* 2021)
Indirect (no physical contact) exposure to dominant individual	Rodents	M			Evidence to support a protective effect of exposure to a dominant individual against depression and anxiety, and a positive effect on exploration and social interaction.	(Brockhurst *et al.* 2015, Ayash *et al.* 2020)
		F			One study found evidence for a more active coping strategy when exposed to a dominant individual. This was characterised by increased exploration, reduced fearfulness, and reduced immobility in a tail-suspension test.	(Lyons *et al.* 2018)
Chronic social stress/social instability stress (repeated exposure to strangers)	Rodents	M			Some evidence to support negative effects of social instability stress, although these are not necessarily permanent.	(McCormick *et al.* 2008, Sterlemann *et al.* 2008, Green *et al.* 2013, dos Santos Guilherme *et al.* 2022)
		F			Two studies showed negative effects of social instability stress, including increased depression, anxiety, and impaired social interactions. However, one study found reduced anxiety in females in oestrus.	

(Grey-shaded cells highlight primate studies; S = sex (F = female, M = male, B = both/mixed, ? = not stated)

In summary, social stress can cause a range of negative effects and can also be contagious, as exposure to stressed rats can cause similar effects to direct stress. However, milder and less direct forms of social stress may have an ‘inoculating’ effect, helping to promote a more active coping strategy, reduce depression and anxiety, and increase exploration and social interaction. Therefore, exposing individuals to mildly challenging social situations may be beneficial, as long as less dominant individuals are not being subjected to prolonged or repeated aggression from more dominant conspecifics. Orangutans are already likely to encounter some degree of social challenge during their time in rehabilitation, for example being moved to an enclosure with unfamiliar conspecifics as infants. However, any social stress is either likely to be one-off (e.g. the initial experience of encountering unfamiliar orangutans) or chronic (e.g. ongoing competition within the enclosure group), rather than the brief, regular, and time-constrained social stressors described in this section. As young orangutans and adult female orangutans in the wild are more likely to associate with related females, particularly their mothers (Ashbury *et al.* 2020), it may be stressful to be in prolonged proximity to unrelated orangutans, especially males (Kunz *et al.* 2021). Therefore, it would be beneficial to test the effects of a more controlled programme of mild social stress. In addition, the potential dynamics of stress contagion, balanced with the therapeutic effects of conspecifics (see *Prolonged isolation*), should be explored further, to identify potential benefits while minimising further harm.

### Exercise

Building locomotion skills is an important aspect of orangutan rehabilitation, as orangutans must be cognitively and physically capable of traversing the complex forest environment (Thorpe *et al.* 2007; Tecwyn 2013; Halsey *et al.* 2017). However, locomotion can also have beneficial psychological side-effects as a form of exercise. There is strong evidence that voluntary wheel-running in rodents can protect against stress, and additional evidence that forced exercise can be beneficial in some situations (see Table 2[e]). Zhang *et al.* (2021) found that mice with high baseline levels of physical activity, measured by voluntary wheel-running, that were subjected to social defeat stress were more sociable in a social interaction test than subjects with low levels of physical activity. Voluntary exercise is preferable for several reasons, including ethical ones, and Leasure and Jones (2008) found that forced exercise increased anxiety. This suggests that a lack of agency is constraining the potential benefits of exercise in this context. However, these negative results contrast with Greenwood *et al.* (2003), who found that both forced and voluntary exercise in rats improved response to fear conditioning and escape in a shuttle-box test. The authors explain this as being due to the experimental apparatus: instead of a treadmill, a wheel was designed to simulate a natural stop-start running pattern and distance that closely resembled voluntary wheel-running. As well as replicating a more natural style of movement for rats, this apparatus also replicates the ‘stop/start’ structure of high intensity interval training in humans. Interval training, where exercise involves short bursts of intense activity alternated with periods of recovery, is thought to help generate fitness and improve cardiac health in humans more quickly than prolonged ‘endurance’ periods of moderate exercise (Wisløff *et al.* 2009; Gillen & Gibala 2014), as well as having benefits for psychological well-being (Martland *et al.* 2022). For orangutans, species-appropriate exercise may involve guided exercise which simulates natural physical activities such as travelling through the canopy, gap-crossing, or bending and breaking branches for nest-building. However, providing them with any opportunity or reason to move will encourage physical activity.

**Table 2(e).** Summary of exercise treatment effects by taxonomic group, and sex (21/266 papers reviewed)

Treatment	Taxonomic group	S	E	R	Notes on treatment	Most relevant studies
Voluntary exercise *(wheel running)*	Rodents	M			Evidence to support beneficial effect of voluntary wheel running in protecting against stress when exposed to later stressors, particularly in individuals with high baseline levels of physical activity. However, two studies report negative effects on anxiety in male rats. One study found improved fear extinction and reduced fear renewal in males, but not females.	(Dishman *et al.* 1997, Greenwood *et al.* 2003, 2005, 2007, 2012, Leasure & Jones 2008, García-Capdevila *et al.* 2009, Fuss *et al.* 2010, Bouchet *et al.* 2017, Mul *et al.* 2018, Robinson *et al.* 2019, Tanner *et al.* 2019, Zhang *et al.* 2021, Calpe-López *et al.* 2022)
		F			Low number of studies, but evidence to support beneficial effect of voluntary wheel running in protecting against future stress. One study found improved active coping strategies in females, but not males.	
Forced exercise *(treadmill/* *wheel running)*	Rodents	M			Evidence to support a protective effect of forced exercise against anxiety, depression, and spatial memory.	(Fulk *et al.* 2004, Leasure & Jones 2008, Greenwood *et al.* 2013, Lalanza *et al.* 2015, Kochi *et al.* 2017, Pietrelli *et al.* 2018, Yan *et al.* 2023)
		F			Conflicting evidence, effect on resilience is unclear.	

(Grey-shaded cells highlight primate studies; S = sex (F = female, M = male, B = both/mixed, ? = not stated)

The results in Table 2(e) indicate that species-specific, voluntary exercise may have numerous benefits, particularly in protecting against the effects of future stress and recovering from past stress. ‘Forest school’, where infant orangutans are taken into the forest by human caretakers to learn a variety of different skills, will already involve a certain amount of physical activity (Preuschoft *et al.* 2021). This is arguably the best opportunity for exercise prior to pre-release islands or living in the wild, in combination with large, complex enclosures that provide plenty of opportunities for locomotion. Although many orangutans will learn by trial and error, some less active individuals may require more encouragement to engage in physical activity. Social learning could be utilised here, as well as potential solutions such as using ropes to pull food higher into the canopy to encourage climbing. In certain circumstances, human caretakers have themselves learned tree-climbing skills to encourage orangutans to climb to higher levels in the canopy (Epstein & Reed 2019). However, there is a wide variety of forest school capacity and enclosure sizes among different rehabilitation centres, and not all centres have access to pre-release islands. Therefore, careful consideration should be given to the provision of in-cage physical enrichment and roof feeding (Chappell & Thorpe 2022), and, in the longer term, building larger enclosures.

## Discussion

In order to effectively integrate resilience interventions into orangutan rehabilitation programmes, it is essential to strike the right balance, by exposing orangutans to opportunities where they can build resilience and prepare for challenges in the wild, while protecting them against further harm. In this review, resilience themes have been drawn from human and animal interventions, and these interventions have been assessed in terms of their potential to be adapted for orangutan rehabilitation centres.

### Study limitations

Although this scoping review has spanned multiple disciplines, from animal biology to human psychology, the results have been limited by the search terms used. As these terms centred around ‘resilience’ and ‘stress’, the search may have missed related topics which do not use either of these terms. In addition, as the results show such a diversity of different aspects and expressions of resilience, each of the themes described above could have their own scoping review in terms of relevance for orangutan rehabilitation.

Due to the diversity of species included in this review, from mice to humans, caution must be applied in extrapolating these findings to orangutans. For example, social stress in close-knit, hierarchical taxonomic groups such as mice and rats may be expressed in different ways to orangutans, who are semi-solitary in the wild, living in dispersed societies and only forming temporary aggregations (Galdikas 1985; Malone *et al.* 2012; Roth *et al.* 2020). In addition, most of the animal research presented here is carried out in a laboratory environment, with human-manipulated genetics, where life histories of the research subjects are already known. These conditions are very different to those in orangutan rehabilitation centres, where life histories are rarely known, and orangutans are likely to have had complex life experiences which intrinsically affect resilience. Despite these limitations, the fact that the animal resilience treatments all relate to at least one human resilience theme indicates that there are aspects of resilience which bridge these taxa, despite clear species differences.

In terms of the findings on human resilience interventions, some of these themes may be easier to translate to orangutan rehabilitation centres than others. For example, interventions such as problem-solving, social interactions, and physical activity are relatively straightforward to use as a measure of resilience and/or apply as an intervention in orangutans. In addition, the animal resilience literature indicates that these aspects can be influenced through practical interventions. However, other attributes relevant to human resilience, such as emotional intelligence, self-awareness, and a sense of agency, are more difficult to identify and measure, let alone ‘teach’, through non-linguistic means. However, there is plenty of scope here for investigating treatments which utilise one or more of the human and/or animal themes to foster or measure resilience in the context of orangutan rehabilitation.

### Main findings

A recurring theme in this review is that mild and manageable interventions are the safest form of resilience treatment. Benefits were found from interventions that involved short-term and/or manageable forms of stress, including brief separations, mildly frustrating cognitive enrichment, and opportunities to indirectly navigate difficult social interactions. Many of these can be integrated into existing management schedules as part of enrichment provision at rehabilitation centres.

However, there are potential conflicts between different types of stress-related interventions. For example, although mild stress inoculation appears to be beneficial, the possible U-shaped relationship between lifetime stressors and resilience should be considered. There may be a stress ‘threshold’ for orangutans that would be nearly impossible to identify, considering the frequently unknown life histories of orangutans in rehabilitation. Research into this topic might be easier to conduct in a zoo environment, where there are records of entire life histories and major stressors, such as moving to a new zoo. Although orangutans will be exposed to stressful experiences in the wild, they will usually have some opportunity to escape or avoid such situations. As there is limited ability for captive orangutans to control or avoid harms, it is important to limit their exposure as much as possible to potentially traumatic events which they do not have the opportunity to escape. In humans, potentially traumatic events are those which are perceived by the individual as having a real or potential threat to the life or bodily integrity of the self or others (American Psychiatric Association 2013; National Child Traumatic Stress Network [NCTSN] 2023). In the rehabilitation setting, examples of potentially traumatic events may include orangutans witnessing conspecifics being darted for a veterinary procedure, physical threats or attacks from conspecifics in an enclosed space or being confined in a box while being transported to a pre-release island. Peer support may also help orangutans to support one another during stressful experiences. However, more research needs to be carried out on stress contagion and the potential positive and negative effects of housing more stressed individuals with less stressed individuals. This type of arrangement may be beneficial for the more stressed individual but could lead to the negative effects of stress contagion for their conspecific.

Bearing in mind the potential risks of introducing stressful experiences, any investigation of these kinds of interventions should begin with the mildest form, building from this if found to be appropriate and effective. Cognitively demanding challenges through enrichment may be an appropriate way of providing opportunities to overcome controllable frustration and foster independence without causing harm. There are many different forms of enrichment that can be provided to primates, including food-based, occupational (including cognitive), structural, sensory, and social (Bloomsmith *et al.* 1991), and each of these categories has the potential to address a number of different factors contributing to resilience. Clark (2011) highlights the importance of maintaining interest and long-term engagement in enrichment, ensuring that the level of complexity is appropriate for the individual, and rotating or modifying enrichment at regular intervals. Physical enrichment is also important, as voluntary exercise can have numerous benefits and protective effects against future stress. Regarding opportunities for exercise, further research is needed into the extent to which orangutans of different age groups have access to forest school, and the frequency/duration of forest school sessions.

In addition to adding enrichment to an enclosure, the design of the enclosure itself is something that can be enriched and modified to encourage cognitive and physical activity, for example by using the Enclosure Design Tool to promote wild-type behaviours (Thorpe *et al.* 2022). Enrichment provision spans a large proportion of the human resilience themes identified in this review, including optimism/positive affect, exercise/physical activity, cognitive skills, agency/independence, problem-solving, planning, persistence, hardiness, goal orientation, and flexibility. However, the findings of this review indicate that long-term planning is essential to ensure that later exposure to less-enriched conditions does not reverse the benefits. A negative change in environmental conditions may have a severely detrimental effect on resilience: for example, during the change from forest school to enclosed environments, or moving an orangutan back into an enclosure after living on a pre-release island before release into the wild. It would be useful to monitor the effectiveness of existing enrichment methods and explore the relationship between enrichment and optimism/pessimism in orangutans.

Some studies identified a role of resilient personality traits, for example low anxiety, low emotionality, and positive affect. This makes sense in terms of the wider literature on animal personality, where research indicates that personality traits can influence cognitive styles (the way information is acquired and processed), strategies for balancing risk and reward, and well-being (Capitanio 2011; Sih & Del Giudice 2012; Cole & Quinn 2014; Zandberg *et al.* 2017). One study on chimpanzees found that personality traits accounted for around 50% variance in well-being, with ‘extraversion’, ‘agreeableness’ and ‘low neuroticism’ being particularly relevant (King & Landau 2003). The importance of personality is also seen in a study of 300 captive gorillas (*Gorilla gorilla gorilla*), which found that extraversion was associated with longer lifespans (Weiss *et al.* 2013). Therefore, it is likely that individual orangutans will have different resilience ‘baselines’ and may respond to resilience interventions in different ways depending upon their personality traits. Further research is needed into expected resilience levels in orangutans, and individual variation in coping styles, in order to be able to measure the effectiveness of interventions.

### Animal welfare implications

Since so little is known about great ape resilience, it is difficult to know where to start with more ‘risky’ interventions like stress inoculation, which might have the potential to cause further harm. It might be argued that the risks outweigh any potential benefits to orangutans. In zoo environments, for example, where the main consideration for caretakers is the well-being of the animal, deliberately causing stress or frustration might be seen as unacceptable. However, there is a strong justification for investigating these types of resilience treatments, as long as a principle of caution is applied throughout the process. For orangutans that are likely to be returned to the wild, the justification is clear: these individuals will face unavoidable stress and new challenges, probably daily, and preparing them for this reality is essential for their ability not only to cope, but to thrive in their new environment. Although rehabilitation centres strive to teach essential life skills to the orangutans in their care, some challenges will be experienced for the first time after orangutans are released into the wild. For example, orangutans in rehabilitation receive a balanced, regular diet to maximise their well-being in captivity (Schmidt 2004). However, due to the temporal availability of food in the wild due to tree masting (mass fruiting) events, wild orangutans experience a dramatic fluctuation of weight loss and gain (Knott 1998). Having prior experience of manageable stress and frustration will help orangutans develop the persistence and hardiness to endure this kind of difficult experience, and the flexibility and problem-solving skills to overcome them. However, orangutans and other great apes in lifelong captivity, in environments like zoos, can also benefit from the experience of overcoming challenging situations. As well as general stressors associated with zoos (Hosey 2000; Birke 2002; Skynner *et al.* 2004), great apes are also likely to experience major challenges throughout their life, including moving to a new zoo, welcoming a new group member into their enclosure, construction/maintenance work, and medical treatment. Therefore, resilience is important for captive great apes to be able to bounce back from these stressors and maximise their well-being.

It is apparent from these findings that there is substantial scope for further research into understanding great ape resilience and the potential benefits of resilience interventions. More work needs to be done to develop and test individual interventions in each of the areas mentioned above, tailoring each intervention to maximise their effectiveness in captive environments, and trialling different combinations of interventions. Ultimately, the purpose of these investigations would be to prepare for the implementation of a long-term study, to trial and measure the effect of a resilience intervention programme on the survival rate of orangutans released back into the forest. This could act as an essential resource for rehabilitation centres in helping to prepare orangutans as much as possible during their time in rehabilitation and inform decisions as to when each individual is ready to face independent life in the wild.

## Conclusion

This review has identified key overarching themes within the human and animal resilience literature and has critically analysed the applicability of different treatments within the context of orangutan rehabilitation. Several starting points have been suggested, with the caveat that further research is necessary into each of these potential treatments and a principle of caution should be applied. In general, the interventions which offer a mild, but manageable, challenge appear to be the most effective and appropriate in the context of orangutan rehabilitation. Due to the lack of existing knowledge about non-human great ape resilience, it is essential that there is a foundation from which effective interventions can be developed. Therefore, this review aims to be a starting point for future research into this essential field, with implications not only for the survival of orangutans released into the wild, but also for the well-being of great apes in all captive environments.

## Supporting information

Bridgeland-Stephens et al. supplementary material Bridgeland-Stephens et al. supplementary material

## Supplementary material

The supplementary material for this article can be found at http://doi.org/10.1017/awf.2023.97.
