# Supplementary material for: Potential resilience treatments for orangutans (Pongo spp.): Lessons from a scoping review of interventions in humans and other animals
Source: Anim Welf. 2023 Dec 4;32:e77. doi: 10.1017/awf.2023.97 (PMC10937215; doi:10.1017/awf.2023.97)
Supplement: Bridgeland-Stephens et al. supplementary material [file S0962728623000970sup001.pdf]

S1 Appendix. Glossary of common terms

**Term**	**Description**
Acute stress	Short-term stress, often relatively intense. In the context of this review, acute stress occurs within a single day, often combining multiple forms of stressor within one session.
Agency	Proactive and flexible engagement with the environment beyond immediate needs to promote individual well-being (1).
Chronic stress	Stress that occurs over a period of time. In the context of this review, chronic stress is categorised as occurring over at least ten days.
Cognitive skills	Skills in cognitive processes related to sensory input, e.g. learning, memory, and causal reasoning (2).
Elevated platform stress	A rodent stress protocol involving placing subjects on an elevated platform in a brightly-lit environment (e.g. 3).
Emotional intelligence	The capacity to make intelligent use of emotional information (4).
Flexibility	The ability to adapt to different kinds of situations and challenges.
Hardiness	A combination of self-control, persistence, and self-motivation to overcome challenges (5).
Learned helplessness	The inability to avoid stressor(s), having experienced inescapable stress (6).
Meditation	An umbrella term for various methods of regulating focus and emotions (7).
Mindfulness	The practice of intentionally maintaining focus on the present moment in a non-judgmental way (8).
Optimism/pessimism	The tendency to believe that future events will be favourable/unfavourable (9).
Positive psychology	Psychological perspective which emphasises helping patients to flourish, rather than focusing only on pathology (10).
Positive/ negative affect	Positive/negative emotional state.
Self-awareness, self-reflection	The capacity/activity of being aware of one's own thoughts, behaviour, emotions, and influences.
Self-confidence/ self-esteem	Positive conceptualisation and feelings about oneself (11).
Self-control/ behavioural inhibition	The ability to self-regulate behaviour, for example to delay gratification.
Social defeat	Subordination by a dominant, usually physically larger, intruder of the same species (e.g. 12).
Stress inoculation	Exposure to manageable stress leading to resistance against future stress (13,14).

References for S1 Appendix

1. Špinka M, Wemelsfelder F. Environmental Challenge and Animal Agency. In: Animal Welfare. Wallingford, UK: CAB International; 2011. p. 27–44.

2. Clark FE. Great ape cognition and captive care: Can cognitive challenges enhance well-being? Appl Anim Behav Sci. 2011;135:1–12.
3. Yang Y, Cao J, Xiong W, Zhang J, Zhou Q, Wei H, et al. Both stress experience and age determine the impairment or enhancement effect of stress on spatial memory retrieval. J Endocrinol. 2003;178(1):45–54.
4. Armstrong AR, Galligan RF, Critchley CR. Emotional intelligence and psychological resilience to negative life events. Pers Individ Dif. 2011 Aug;51(3):331–6.
5. Maddi SR. Hardiness: the courage to grow from stresses. J Posit Psychol. 2006;1(160–168).
6. Overmier JB, Seligman ME. Effects of Inescapable Shock Upon Subsequent Escape and Avoidance Responding. J Comp Physiol Psychol. 1967;63(1):28–33.
7. Lutz A, Slagter HA, Dunne JD, Davidson RJ. Attention regulation and monitoring in meditation. Trends Cogn Sci. 2008 Apr 1;12(4):163–9.
8. Las Hayas C, Izco-Basurko I, Fullaondo A, Gabrielli S, Zwiefka A, Hjemdal O, et al. UPRIGHT, a resilience-based intervention to promote mental well-being in schools: Study rationale and methodology for a European randomized controlled trial. BMC Public Health. 2019 Oct 29;19(1):1–10.
9. Forgeard MJC, Seligman MEP. Seeing the glass half full: A review of the causes and consequences of optimism. Prat Psychol. 2012 Jun;18(2):107–20.
10. Seligman MEP, Csikszentmihalyi M. Positive psychology: An introduction. Am Psychol. 2000;55(1):5–14.
11. Mandleco BL, Peery JC. An organizational framework for conceptualising resilience in children. J Child Adolesc Psychiatr Nurs. 2000;13(3):99–111.
12. Gardner KL, Thrivikraman K V., Lightman SL, Plotsky PM, Lowry CA. Early life experience alters behavior during social defeat: Focus on serotonergic systems. Neuroscience. 2005;136(1):181–91.
13. Parker KJ, Buckmaster CL, Schatzberg AF, Lyons DM. Prospective investigation of stress inoculation in young monkeys. Arch Gen Psychiatry. 2004 Sep 1;61(9):933–41.
14. Meichenbaum D, Cameron R. Stress inoculation training: Toward a general paradigm for training coping skills. In: Meichenbaum D, Jaremko M, editors. Stress Reduction and Prevention. New York: Plenum; 1983. p. 115–54.

S2 Appendix. Overview of common tools to measure resilience in humans

Category	Name of measure	Citation
Resilience	Adult Resilience Scale	(1)
	Adult Resilience Social Acceptability Measure	(2)
	Brief Resilience Scale	(3)
	Child and Youth Resilience Measure	(4)
	Connor-Davidson Resilience Scale	(5)
	Coping Inventory for Stressful Situations	(6)
	Coping Orientations to Problems Experienced (COPE) Scale	(7)
	Coping Responses Inventory	(8)
	Dispositional Resilience Scale	(9)
	Distress Tolerance Scale	(10)
	MeQuilibrium Resilience Measure	(11)
	Resilience at Work (R@W) Scale	(12)
	Resilience Scale	(13)
	Resilience Scale for Adolescents (READ-28)	(14)
	Resilience Scale for Adults	(15)
	Strategic Approach to Coping Scale	(16)
	Utrecht Coping List	(17)
	Ways of Coping Checklist	(18)
Stress	Brief Symptom Inventory (Global Distress)	(19)
	Depression, Anxiety, and Stress Scale	(20)
	Perceived Stress Questionnaire	(21)
	Perceived Stress Scale	(22)
	PTSD Checklist	(23)
	PTSD Symptom Scale - Self-Report	(24)
	Symptoms of Stress Inventory	(25)
	Tension Thermometer	(26)
Emotional regulation	Cognitive Emotion Regulation Questionnaire	(27)
	Difficulties in Emotion Regulation Scale	(28)
	Emotion Regulation Questionnaire	(29)
	Implicit Theories of Emotion Scale	(30)
	Proneness to Provocation	(31)
	Strengths and Difficulties Questionnaire - Emotional Problems Score	(32)
	Self-monitoring of anger reactions	(33)
	State-Trait Anger Expression Inventory	(34)
Depression	Beck Depression Inventory	(35)
	Center for Epidemiologic Studies Depression Scale	(36)
	Depression, Anxiety, and Stress Scale	(20)

Category	Name of measure	Citation
	Mini International Neuropsychiatric Interview (MINI) Depression Scale	(37)
	Patient Health Questionnaire (for depression)	(38)
	Patient-Reported Outcomes Measurement Information System (PROMIS) Depression and Anxiety symptom measures	(39)
	Reynolds Adolescent Depression Scale	(40)
Anxiety	AN-UD Anxiety Scale	(41)
	Cognitive-Somatic Anxiety Questionnaire	(42)
	Depression, Anxiety, and Stress Scale	(20)
	Generalised Anxiety Disorder Scale	(43)
	Patient-Reported Outcomes Measurement Information System (PROMIS) Depression and Anxiety symptom measures	(39)
	State-Trait Anxiety Inventory Trait Anxiety Scale (STAI A-TRAIT)	(44)
Psychological wellbeing	Mental Health Continuum	(45)
	PERMA Profiler	(46)
	Positive and Negative Affect Scale (PANAS)	(47)
	Psychological Well-Being Scale	(48)
	Warwick-Edinburgh Mental Wellbeing Scale	(49)
Personality	ABCD-M personality test	(50)
	Formal Characteristics of Behaviour - Temperament Inventory	(51)
	Revised NEO Personality Inventory	(52)
	Ten-Item Personality Inventory	(53)
Self-efficacy	Coping Self-Efficacy	(54)
	General Self-Efficacy Scale	(55)
	Internal Control Index	(56)
	Self-Efficacy Scale	(57)
Self-esteem	Coopersmith Self-Esteem Inventory	(58)
	Rosenberg Self-Esteem Scale	(59)
	State Self-Esteem Scale - Current Thoughts	(60)
Mindfulness	Five Facet Mindfulness Questionnaire	(61)
	Freiburg Mindfulness Inventory	(62)
Other	Acceptance and Action Questionnaire	(63)
	Achievement Goal Scale	(64)
	Cognitive Fusion Questionnaire	(65)
	Fear Survey Schedule	(66)
	Igroup Presence Questionnaire	(67)
	Interpersonal Support Evaluation List	(68)
	Life Orientation Test (for optimism)	(69)
	Self-Compassion Scale	(70)
	Social Adjustment Scale	(71)

References for S2 Appendix

1. Mackrain M. Deveraux Adult Resilience Scale. Lewisville, North Carolina: Kaplan Early Learning Company Publishing; 2008.
2. Barrett PM, Lowry-Webster HM, Turner CM, Johnson. The FRIENDS program integrity checklists and social validity measures. (Unpublished manuscript) School of Applied Psychology, Griffith University, Queensland, Australia. 1998.
3. Smith BW, Dalen J, Wiggins K, Tooley E, Christopher P, Bernard J. The brief resilience scale: assessing the ability to bounce back. Int hournal Behav Med. 2008;15(3):194–200.
4. Ungur M, Liebenberg L. Assessing Resilience Across Cultures Using Mixed Methods: Construction of the Child and Youth Resilience Measure. J Mix Methods Res. 2011;5(2):126–49.
5. Connor KM, Davidson JRT. Development of a new resilience scale: the Connor-Davidson Resilience Scale (CD-RISC). Depress Anxiety. 2003;18(2):76–82.
6. Endler NS, Parker JDA. Coping Inventory for Stressful Situations (CISS): Manual. Toronto: Multi-Health Systems; 1990.
7. Carver CS, Scheier MF, Weintraub JK. Assessing coping strategies: A theoretically based approach. J Pers Soc Psychol. 1989;5:267–83.
8. Moos RH. Coping Responses Inventory: CRI from Adults. Odessa, Texas: Psychological Assessment Resources; 1993.
9. Bartone P. Development and validation of a short hardiness measure. In: Annual Convention of the American Psychological Society. Washington DC; 1991.
10. Simons J, Gaher R. The Distress Tolerance Scale: development and validation of a self-report measure. Motiv Emot. 2005;29:83–102.
11. Shatté A, Perlman A, Smith B, Lynch WD. The Positive Effect of Resilience on Stress and Business Outcomes in Difficult Work Environments. J Occup Environ Med. 2017;59(2):135–40.
12. Winwood PC, Colon R, Psych O, Mcewen K. A Practical Measure of Workplace Resilience Developing the Resilience at Work Scale. 2013;
13. Wagnild GM, Young H. Development and psychometric evaluation of the resilience scale. J Nurs Meas. 1993;1(2):165–78.
14. Hjemdal O, Friborg O, Stiles TC, Martinussen M, Rosenvinge JH. A New Scale for Adolescent Resilience: Grasping the Central Protective Resources Behind Healthy Development. Meas Eval Couns Dev. 2006;39(2):84–96.
15. Friborg O, Hjemdal O, Rosenvinge JH, Martinussen M. A new rating scale for adult resilience: what are the central protective resources behind healthy adjustment? Int J Methods Psychiatr Res. 2003;12(2):65–76.
16. Hobfoll SE, Dunahoo CL, Ben-Porath Y, Monnier J. Gender and coping: The The dual-axis model of coping. Am J Community Psychol. 1994;22:49–82.
17. Schreurs PJG, van de Willige G, Tellegen B, Brosschot JF. Herziene handleiding Utrechtse Coping Lijst (UCL). Lisse: Swets & Zeitlinger B.V.; 1993.
18. Nakano K. The Role of Coping Strategies on Psychological and Physical Well-being. Jpn Psychol Res. 1991;33(4):160–7.
19. Derogatis LR. Brief Symptom Inventory 18: Administration, scoring and procedures manual. Minneapolis, Minnesota: NCS Pearson; 2001.
20. Lovibond SH, Lovibond PF. Manual for the Depression Anxiety & Stress Scales. 2nd Editio.

Sydney: Psychology Foundation; 1995.
21. Levenstein S, Prantera C, Varvo V, Scribano ML, Berto E, Luzi C, et al. Development of the perceived stress questionnaire: A new tool for psychosomatic research. J Psychosom Res. 1993 Jan 1;37(1):19–32.
22. Cohen S, Kamarck T, Mermelstein R. A Global Measure of Perceived Stress. J Health Soc Behav. 1983;24(4):385–96.
23. Weathers FW, Litz BT, Keane TM, Palmieri PA, Marx BP, Schnurr PP. The PTSD Checklist for DSM-5 (PCL-5) [Internet]. National Center for PTSD. 2013. Available from: www.ptsd.va.gov
24. Foa EB, Riggs DS, Dancu C V., Rothbaum BO. Reliability and validity of a brief instrument for assessing post-traumatic stress disorder. J Trauma Stress. 1993 Oct 1;6(4):459–73.
25. Leckie MS. Symptoms of Stress Inventory. Seattle, Washington: University of Washington Press; 1979.
26. Long BC. Aerobic conditioning and stress inoculation: A comparison of stress-management interventions. Cognit Ther Res. 1984;8(5):517–41.
27. Garnefski N;, Kraaij V. Cognitive emotion regulation questionnaire-development of a short 18-item version (CERQ-short). Pers Individ Dif. 2006;41:1045–53.
28. Gratz KL, Roemer L. Multidimensional assessment of emotion regulation and dysregulation: development, factor structure, and initial validation of the difficulties in emotion regulation scale. J Psychopathol Behav Assessment2. 2004;26:41–54.
29. Gross JJ, John OP. Individual differences in two emotion regulation processes: implications for affect, relationships, and well-being. J Pers Soc Psychol. 2003 Aug;85(2):348–62.
30. Tamir M, John OP, Srivastava S, Gross JJ. Implicit Theories of Emotion: Affective and Social Outcomes Across a Major Life Transition. J Pers Soc Psychol. 2007;92(4):731–44.
31. Novaco RW. Anger Control: The Development and Evaluation of an Experimental Treatment. Lexington: Lexington Books; 1975.
32. Goodman R. The strengths and difficulties questionnaire: A research note. J Child Psychol Psychiatry Allied Discip. 1997 Jul;38(5):581–6.
33. Mahoney MJ, Thoresen CE. Self-Control: Power to the Person. Monterey, California: Brooks-Cole; 1974.
34. Spielberger CD. Manual for the State-Trait Anger Expression Inventory (STAXI). Odessa, Florida: Psychological Assessment Resources; 1988.
35. Beck AT, Ward CH, Mendelson M, Mock J, Erbaugh J. An inventory for measuring depression. Arch Gen Psychiatry. 1961;4(6):561–71.
36. Radloff LS. The CES-D Scale: A self-report depression scale for research in the general population. Appl Psychol Meas. 1977;1(3):385–401.
37. Lecrubier Y, Sheehan D V., Weiller E, Amorim P, Bonora I, Sheehan KH, et al. The Mini International Neuropsychiatric Interview (MINI). A short diagnostic structured interview: reliability and validity according to the CIDI. Eur Psychiatry. 1997 Jan 1;12(5):224–31.
38. Kroenke K, Spitzer RL, Williams JBW. The PHQ-9: Validity of a Brief Depression Severity Measure. J Gen Intern Med. 2001;16(9):606.
39. Pilkonis PA, Choi SW, Reise SP, Stover AM, Riley WT, Cella D. Item Banks for Measuring Emotional Distress From the Patient-Reported Outcomes Measurement Information System (PROMIS®): Depression, Anxiety, and Anger. Assessment. 2011;18(3):263–83.
40. Reynolds WM. Reynolds Adolescent Depression Scale: Professional Manual. Odessa, Florida: Psychological Assessment Resources; 1987.
41. Kozina A. The LAOM Multidimensional Anxiety Scale for Measuring Anxiety in Children

and Adolescents: Addressing the Psychometric Properties of the Scale. J Psychoeduc Assess. 2012 Dec 5;30(3):264–73.
42. Schwartz GE, Davidson RJ, Goleman DJ. Patterning of cognitive and somatic processes in the self-regulation of anxiety: Effects of meditation versus exercise. Psychosom Med. 1978;40(4):321–8.
43. Spitzer RL, Kroenke K, Williams JBW, Löwe B. A brief measure for assessing generalized anxiety disorder: the GAD-7. Arch Intern Med. 2006 May 22;166(10):1092–7.
44. Spielberger CD, Gorsuch RL, Lushene RE. STAI Manual for the State-Trait Anxiety Inventory. Palo Alto: Consulting Psychologists Press; 1970.
45. Keyes CLM. The Mental Health Continuum: From Languishing to Flourishing in Life. J Health Soc Behav. 2002;43(2):207–22.
46. Butler J, Kern ML. The PERMA-profiler: A brief multidimensional measure of flourishing. Int J Wellbeing. 2016;6(3):1–48.
47. Watson D, Clark LA, Tellegen A. Development and validation of brief measures of positive and negative affect: The PANAS scales. J Personal Soc Psychol. 1988;54(6):1063–70.
48. Ryff CD. Happiness is everything, or is it? Explorations on the meaning of psychological well-being. J Personal Soc Psychol. 1989;57:1069–81.
49. Tennant R, Hiller L, Fishwick R, Platt S, Joseph S, Weich S, et al. The Warwick-Edinburgh Mental Wellbeing Scale (WEMWBS): development and UK validation. Health Qual Life Outcomes. 2007;5(63).
50. Minulescu M. ABCD-M Scales Description. In: ABCD-M Technical Manual (in Romanian). Sinapsis Publishing; 2008. p. 87–116.
51. Strelau J, Zawadzki. The Formal Characteristics of Behavior - Temperament Inventory (FCB-TI): theoretical assumptions and scale construction. Eur J Pers. 1993;7:313–36.
52. Costa PT, McCrae. The Revised NEO Personality Inventory (NEO-PI-R). In: The SAGE Handbook of Personality Theory and Assessment, Vol 2 Personality Measurement and Testing. 2008. p. 179–98.
53. Gosling SD, Rentfrow PF, Swann WB. A very brief measure of the Big-Five personality domains. J Res Personal. 2003;37:504–28.
54. Chesney MA, Neilands TB, Chambers DB, Taylor JM, Folkman S. A validity and reliability study of the coping self-efficacy scale. Br J Health Psychol. 2006;11:421–37.
55. Schwarzer R, Jerusalem M. Generalized Self-Efficacy Scale. In: Weinman J, Wright S, Johnston M, editors. Measures in health psychology: A user's portfolio Causal and control beliefs. Windsor, England: NFER-NELSON; 1995. p. 35–7.
56. Duttweiler PC. The Internal Control Index: A Newly Developed Measure of Locus of Control. Educ Psychol Meas. 2016 Sep 7;44(2):209–21.
57. Coppel DB. The Relationship of Perceived Social Support and Self-Efficacy to Major and Minor Stressors. University of Washington; 1980.
58. Coopersmith S. SEI Self-Esteem Inventories Manual. Redwood City, California: Mind Garden; 1981.
59. Rosenberg M. Society and the Adolescent Self-Image. Princeton, New Jersey: Princeton University Press; 1965.
60. Heatherton TF, Polivy J. Development and validation of a scale for measuring state selfesteem. J Pers Soc Psychol. 1991;60:895–910.
61. Baer RA, Smith GT, Hopkins J, Krietemeyer J, Toney L. Using self-report assessment methods to explore facts of mindfulness. Assessment. 2006;13:27–45.
62. Buchheld N, Grossman P, Walach H. Measuring Mindfulness in Insight Meditation

(Vipassana) and Meditation-Based Psychotherapy: The Development of the Freiburg Mindfulness Inventory (FMI). J Medit Medit Res. 2001;1:11–34.

63. Hayes SC, Strosahl K, Wilson KG, Bissett RT, Pistorello J, Toarmino D, et al. Measuring experiential avoidance: A preliminary test of a working model. Psychol Rec. 2004 May 23;54(4):553–78.
64. Elliot AJ, Church MA. A hierarchical model of approach and avoidance achievement motivation. J Pers Soc Psychol. 1997;72:218–32.
65. Gillanders D, Bolderston H, Bond FW, Dempster M, Flaxman PE, Campbell L, et al. The development and initial validation of The Cognitive Fusion Questionnaire. Behav Ther. 2014;45(1):83–101.
66. Wolpe J, Lang PJ. A fear survey schedule for use in behaviour therapy. Behav Res Ther. 1964;2(1):27–30.
67. Schubert T, Friedmann F, Regenbrecht H. The experience of presence: Factor analystic insights. Presence Teleoperators Virtual Environ. 2001;10(3):266–81.
68. Cohen S, Memelstein R, Kamarck T, Hoberman H. Measuring the functional components of social support. In: Sarason IG, Sarason B, editors. Social support: Theory, research, and application. The Hague: Martinus Nijhoff; 1985. p. 73–94.
69. Scheier MF, Carver CS. Optimism, coping, and health: Assessment and implications of generalized outcome expectancies. Heal Psychol. 1985;4:219–47.
70. Neff KD. Development and validation of a scale to measure self-compassion. Self Identity. 2003;2:223–50.
71. Weissman MM, Paykel ES. The Depressed Woman: A Study of Social Relationships. Chicago: Universities Press; 1974.

S3 Appendix. Overview of common types of non-human animal behavioural measurements relevant to resilience

Attribute	Relevance	Name of measure	Taxonomic groups
Anxiety	Stress indicator	Elevated plus maze/elevated zero maze	Rodents
		Open field test	Rodents/ agricultural
		Light/dark test	Rodents/birds
		Novelty suppressed feeding test	Rodents
		Behavioural observations	Any
		'Behaviourally anchored' criteria for generalised anxiety disorder.	Primates
Depression	Stress indicator	Porsolt forced swim test	Rodents
		Social interaction test (with stranger)	Rodents
		Tail suspension	Rodents
		Latency to escape electric shock.	Rodents/dogs
		Sucrose consumption test (quantity).	Rodents
		Sucrose preference test (over water).	Rodents
		Body weight.	Rodents
Memory and spatial learning	Related to problem-solving/ planning	Radial arm maze (win-shift tasks)	Rodents
		Fear conditioning	Rodents
		Two-way shuttle-box	Rodents/dogs
		Morris water maze/Dry Morris water maze (on land)	Rodents
		Spatial foraging task	Birds
		Associative learning task	Birds
		Novel odour recognition memory test	Rodents
		Barnes maze	Rodents
		Y-maze	Rodents
Fear & emotional reactivity	Low emotional reactivity is an aspect of resilience	Acoustic startle	Rodents/ primates
		Two-way shuttle-box	Rodents/dogs
		Fear conditioning/extinction	Rodents
		Tonic immobility test	Birds
		Hole-in-the-wall test	Birds
		Novel object test	Any
Exploration	Curiosity and physical activity are indicators of resilience	Open field test / novel environment	Any
		Novel object	Any
		Novel odour	Rodents
		Novelty-suppressed feeding test	Rodents
		Social exploration/juvenile social exploration	Rodents
		Approach/avoidance test	Primates
		Behavioural observations	Any

Attribute	Relevance	Name of measure	Taxonomic groups
Anxiety	Stress indicator	Elevated plus maze/elevated zero maze	Rodents
		Open field test	Rodents/ agricultural
		Light/dark test	Rodents/birds
		Novelty suppressed feeding test	Rodents
		Behavioural observations	Any
		‘Behaviourally anchored’ criteria for generalised anxiety disorder.	Primates
Inhibition	Inhibitory control is an aspect of resilience	Detour-reaching task	Primates
Other		Behavioural observations (e.g. aggression, vigilance)	Any
		Human-rated personality/behaviour assessments	Dogs
		Locomotion	Any
		Coping profile assessment	Rodents
		Condition of fur	Rodents
		Olfactory discrimination task.	Rodents
		Problem solving digging task.	Rodents
		Vocalisations	Any
